# A Review of Peripheral Artery Disease in Diabetic Patients in Sub-Saharan Africa

**DOI:** 10.7759/cureus.69808

**Published:** 2024-09-20

**Authors:** Ayoyimika O Okunlola, Temitope O Ajao, Abbas Karim, Mwila Sabi, Olayinka Kolawole, Kenneth Ugwoke, Manoj K Mahadevaswamysusheela

**Affiliations:** 1 Internal Medicine, United Lincolnshire Hospitals NHS Trust, Lincolnshire, GBR; 2 General and Acute Medicine, United Lincolnshire Hospitals NHS Trust, Lincolnshire, GBR; 3 Emergency Medicine, United Lincolnshire Hospitals NHS Trust, Lincolnshire, GBR; 4 Respiratory Medicine, United Lincolnshire Hospitals NHS Trust, Lincolnshire, GBR; 5 Internal Medicine, Hull University Teaching Hospitals NHS Trust, Hull, GBR; 6 Vascular Surgery, United Lincolnshire Hospitals NHS Trust, Lincolnshire, GBR; 7 Endocrinology and Diabetes, United Lincolnshire Hospitals NHS Trust, Lincolnshire, GBR

**Keywords:** diabetes mellitus, non-communicable disease, peripheral arterial diseases, re-vascularization, sub-saharan africa

## Abstract

Peripheral artery disease (PAD) is an atherosclerotic occlusive disease of the lower extremities and is associated with an increased risk of amputation and cardiovascular events. The interplay between diabetes and PAD is complex, influenced by shared risk factors such as hypertension, dyslipidemia, and smoking. High rates of undiagnosed diabetes, coupled with barriers to accessing care, contribute to the complexity of managing PAD. Unique to the Sub-Sahara region is associations with communicable diseases such as human immunodeficiency virus and tuberculosis which further complicates the epidemiological landscape. Comprehensive management strategies, including lifestyle modifications, pharmacological interventions, and revascularization procedures, are essential. However, the region faces challenges such as inadequate healthcare infrastructure and high costs of treatment. This narrative review highlights the epidemiology of PAD in people with diabetes, the risk factors associated with PAD, the impact of PAD on the morbidity and mortality of individuals with diabetes, as well as the management of PAD in individuals with diabetes, with attention geared toward Sub-Saharan Africa These insights are critical for developing effective strategies to mitigate the burden of PAD in diabetes, especially in Sub-Saharan Africa. Further research is essential to understand the associations between diabetes and other diseases in the region.

## Introduction and background

Peripheral artery disease (PAD) is a condition characterized by the narrowing or blockage of arteries outside the heart and brain due to atherosclerosis, with the arteries in the lower extremities being the most commonly affected [1]. PAD is associated with an increased risk of lower extremity amputation and is a marker for atherothrombosis in cardiovascular, cerebrovascular, and renovascular beds [1]. While many patients remain asymptomatic or present with atypical symptoms during exertion, about one-third experience intermittent claudication [1,2]. This condition causes aching, cramping, or numbness in the affected limb during physical activity, which subsides with rest. PAD negatively impacts the quality of life and significantly impairs function [2]. Intermittent claudication can lead to reduced walking speed and diminished walking distance, contributing to a gradual decline in function and ultimately resulting in long-term disability [2,3]. In Sub-Saharan Africa, the prevalence of PAD ranges from 3.1% to 24% among individuals 50 years and older, which increases to 39% to 52% among individuals with risk factors [2]. Diabetes mellitus (DM) greatly heightens the risk of PAD and accelerates its progression, making diabetic patients more susceptible to ischemic events and impaired functional status compared to non-diabetic individuals [3]. Diabetic patients with PAD face a higher risk of lower extremity amputation compared to non-diabetic patients, with the risk of amputation four times higher than in the general population [3].

**Figure 1:**
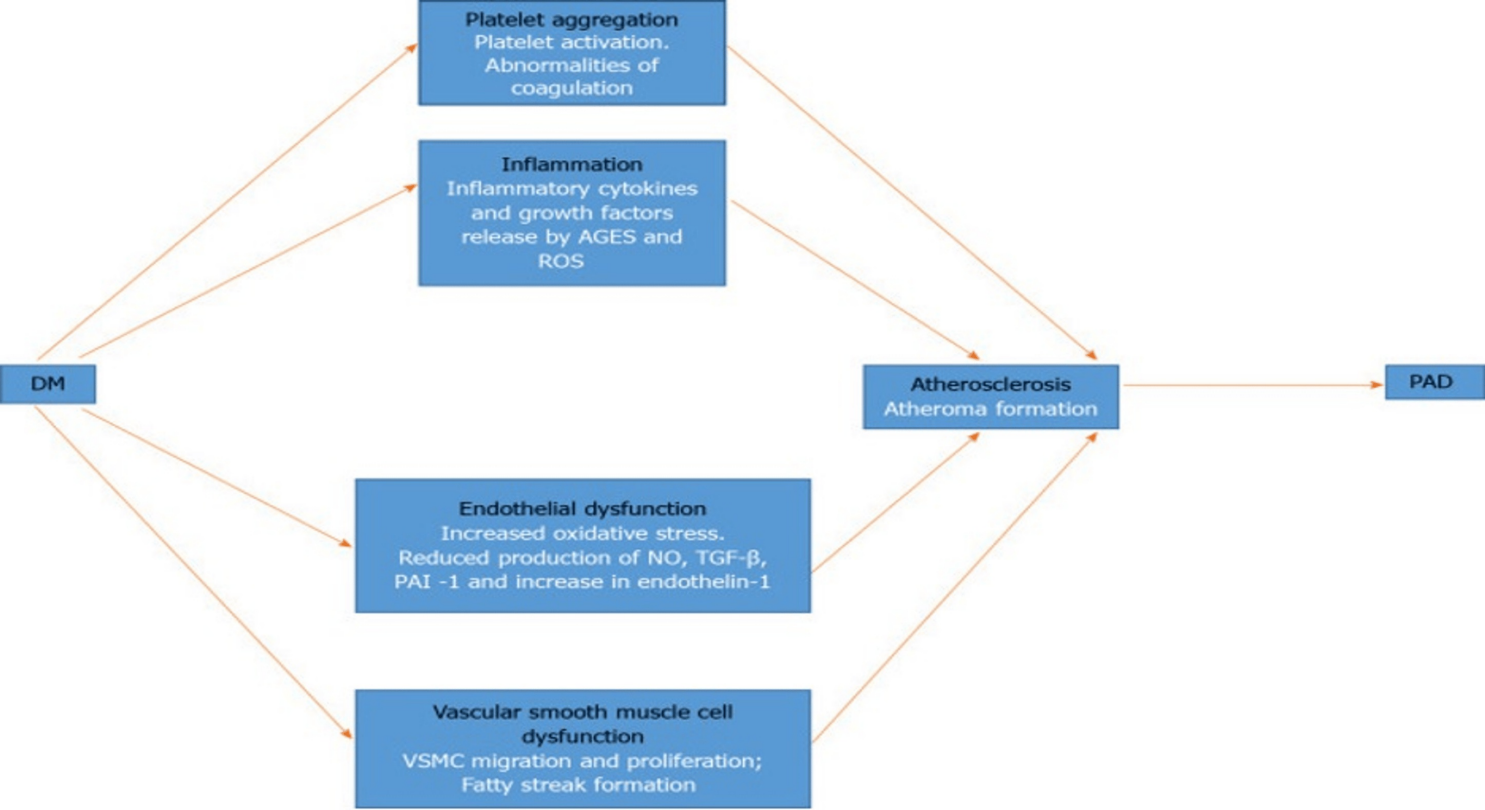
Diagrammatic depiction of the disease mechanisms of peripheral arterial disease (PAD) in diabetes mellitus (DM). Disease processes and their mechanisms are indicated in black and white type, respectively [5]. AGEs: advanced glycation end products; NO: nitric oxide; PAI-1: plasminogen activator inhibitor-1; ROS: reactive oxygen species; TGF-β transforming growth factor-beta; VSMC: vascular smooth muscle cell

Atherosclerosis is a key pathophysiological factor in PAD associated with DM. It commences with atherogenesis and advances to significant blood flow obstruction. In cases of subclinical atherosclerosis, pathological changes can occur before the diagnosis of impaired fasting glucose and DM [4]. In diabetic patients, these changes reflect those seen in other vascular beds. Atherosclerosis is initiated by several pathogenic mechanisms, including endothelial dysfunction, inflammation, platelet aggregation, and vascular smooth muscle cell (VSMC) abnormalities [1]. The mechanism by which this leads to PAD is depicted in Figure 1.

PAD represents a significant yet often under-recognized complication of DM, characterized by the narrowing of peripheral arteries, primarily affecting the legs [1]. In Sub-Saharan Africa, where the surge in non-communicable diseases parallels infectious diseases, the intersection of PAD and diabetes presents unique public health challenges [2].

This narrative review aims to provide a comprehensive overview and synthesis of the existing literature on PAD in diabetic patients in Sub-Saharan Africa. The review focuses on identifying key themes, trends, and gaps in the literature.

## Review

Methodology

A narrative review was conducted to examine the current knowledge on the prevalence, risk factors, and outcomes of PAD in diabetic patients in Sub-Saharan Africa. This review synthesized existing literature to provide a comprehensive understanding of the subject, identifying gaps and offering insights for future research and clinical practice.

Search Strategy

The literature search was performed across several electronic databases, including PubMed, Medline, Google Scholar, and Embase. The search terms included combinations of keywords such as “Peripheral Artery Disease,” “PAD,” “Diabetes Mellitus,” “Sub-Saharan Africa,” “prevalence,” “risk factors,” and “outcomes.” Both medical subject headings (MeSH) and free-text terms were used to broaden the scope of the search. Additionally, relevant articles were identified through hand-searching reference lists of key papers. The search was limited to articles published in the English language to ensure accessibility.

Study Selection

Articles were selected based on their relevance to the below key themes.

Inclusion criteria: Studies discussing the epidemiology of PAD in diabetic patients. Studies reporting risk factors associated with PAD, including demographic, clinical, and socioeconomic factors. Studies discussing the diagnostic methods used to identify PAD in diabetic populations. Studies reporting clinical outcomes and complications related to PAD in diabetic patients, particularly in the context of healthcare access in Sub-Saharan Africa.

Exclusion criteria: Studies focusing solely on populations outside Sub-Saharan Africa or those unrelated to diabetes were excluded. Non-English-language publications, conference abstracts, letters to the editor, and opinion pieces without primary or secondary data were also excluded.

Data Synthesis

A qualitative synthesis of the literature was conducted, summarizing the key findings on PAD prevalence, risk factors, and outcomes among diabetic populations in Sub-Saharan Africa. The review considered both individual country-level studies and multi-country reports to provide a broad perspective. Where available, variations in diagnostic criteria and healthcare infrastructure were discussed.

Epidemiology of diabetes

The number of people worldwide with diabetes grew from 108 million in 1980 to 422 million in 2014, with a global prevalence of 537 million people in 2021 [6]. The prevalence of diabetes across Africa varies and appears to rise with economic development [6,7]. In low-income nations, the prevalence is about 4.4%, increasing to 5.0% in lower-middle-income countries and reaching 7.0% in upper-middle-income countries [6,7]. In 2021, approximately 4.5% of adults aged 20-79 years in Sub-Saharan Africa had diabetes, corresponding to around 24 million people [6]. By 2045, this number is predicted to increase by 129% to 55 million, marking the highest increase among all International Diabetes Federation (IDF) regions. In 2017, the prevalence of diabetes in Sub-Saharan Africa was approximately 6%, with an estimated 15.5 million adults (range = 9.8-27.8 million) living with the disease and associated healthcare costs amounting to USD 3.3 billion [7]. Notably, over 54% of people with diabetes in Sub-Saharan Africa remain undiagnosed, the highest proportion globally [6]. In 2017, the nations with the highest number of people with diabetes (ages 18-99 years) were Ethiopia, South Africa, the Democratic Republic of Congo, Nigeria, and Tanzania. This number is expected to increase by 162.5% by 2045, with an estimated 40.7 million adults suffering from type 2 diabetes and healthcare costs as high as USD 6 billion [8].

Changes in lifestyle and nutrition in Sub-Saharan African populations contribute to the rising prevalence of diabetes, compounded by increased life expectancy in the region [9]. Potential relationships between diabetes and major communicable diseases, such as tuberculosis and human immunodeficiency virus (HIV), further complicate the pattern of increasing diabetes prevalence and pose significant challenges to resource-strained health systems [10]. Additionally, the high prevalence of HIV and the use of antiretroviral therapy (ART) may elevate the risk factors for diabetes, thereby increasing its incidence [11]. ART for HIV, and to a lesser extent HIV itself, is linked to a higher risk of developing metabolic syndrome, which predisposes individuals to type 2 diabetes and cardiovascular disease [11]. The high rates of undiagnosed and uncontrolled diabetes highlight the presence of significant barriers to accessing diagnosis and treatment. The high rates of undiagnosed diabetes suggest that existing screening practices in the region are not effective [10]. Challenges in accessing diagnosis and management include the high financial cost of treatments, especially insulin; limited availability of diagnostic tools, treatments, and glucose monitoring equipment; and the insufficient number and inadequate training of healthcare workers [12,13]. Advancing age as well as a longer duration of DM were associated with PAD in some studies in Sub-Saharan Africa and elsewhere [14-16].

Prevalence rates of peripheral artery disease among diabetic patients

Identifying the true prevalence of PAD in diabetic patients is challenging, primarily because most patients are asymptomatic and there is a lack of uniformity in PAD screening modalities [1]. PAD is currently screened using various methods, including Doppler-guided ankle-brachial pressure index (ABPI), pulse palpation, waveform analysis, absolute toe pressure, toe brachial pressure index, and transcutaneous oxygen pressure. While each method has its shortcomings, ABPI measurement is the most accurate and consistently reproducible [17]. It has been validated against disease, confirmed angiographically, and is recognized as a non-invasive tool useful for the diagnosis and surveillance of PAD [17]. Additionally, it is a strong marker of generalized atherosclerosis and cardiovascular risk [17]. Globally, the prevalence of PAD in the general population when using ABPI is estimated to be between 20% and 30% [18]. A cross-sectional study involving nearly 10,000 American adults found the prevalence of PAD in people with DM to be 27% [19]. A previous American study reported PAD prevalence rates among symptomatic diabetics at 20% and 29%, respectively [20]. In South Asia, a cohort study found the prevalence to be 24%, while a study in Korea reported a prevalence of 28.7% [21]. A multi-country study on the prevalence and clinical features of PAD in Asian type 2 diabetes patients found a prevalence of 17.7% [22]. However, studies in Karnataka, India, and America reported a lower prevalence of PAD, ranging between 7.5% and 8.5% [19,23]. Conversely, some studies in India and Pakistan found prevalence rates between 30% and 40% [24-26].

In Sub-Saharan Africa, studies on the prevalence of PAD among diabetics are limited. A review by Johnston et al. reported the general prevalence of PAD among diabetic individuals in Sub-Saharan Africa to be between 39% and 52% [2]. Most studies in Nigeria reported PAD prevalence among individuals diagnosed with DM to be between 22% and 52.5% [15,27,28], with one study noting a prevalence as high as 61.7% [22]. Studies in Ghana and Cameroon reported a prevalence of 26.7% and 17%, respectively [29,30]. Two studies in Uganda found prevalence rates of 24% and 39% [31,32], while another study in Ethiopia reported a prevalence of 30% [33]. The differences in prevalence rates can be attributed to variations in diagnostic tools, age of study participants, drug adherence, and management of type 2 DM. Additionally, socioeconomic factors, lifestyle, and duration of diabetes may contribute to these differences. Multiple studies have shown that the duration and severity of diabetes correlate with the incidence and extent of PAD [34-36]. Moreover, glycemic control has emerged as an independent risk factor for PAD with a 1% increment in glycosylated hemoglobin (HbA1c) being associated with a 28% increased risk of PAD development [37].

Risk factors for peripheral artery disease In diabetic patients

Generally, being diabetic increases the risk of developing PAD. Among the diabetic population, markers of poor control are similarly associated with a higher risk [22,38,39]. Individuals with HbA1c levels above 7% have a greater risk of developing PAD [30,36,37]. One study demonstrated that type 2 diabetics requiring both insulin and oral agents are at greater risk as well, with another demonstrating increased risk with oral hypoglycemic agents such as glibenclamide, although this might just be a spurious finding [32,40]. Simply having diabetes for more than 15 years, even with good control, is also a risk factor [40]. The vast majority of research on PAD risk factors is based on Western populations and focuses on traditional cardiovascular risk factors such as age, smoking, dyslipidemia, and hypertension. Numerous studies have demonstrated that individuals over the age of 40 years are at a greater risk of developing PAD in both the general population and specifically among diabetics [22,38,40]. Vascular endothelial damage and subsequent atheroma accumulate with age in all individuals, increasing the risk of vascular phenomena [41].

Smoking is an important lifestyle factor that can independently increase the risk of PAD [39,40,42]. In the general population, smoking has been identified as a potent risk factor for PAD, doubling the risk of disease development [22,41]. In a study done in Sub-Saharan Africa, the odds of developing PAD in patients with type 2 DM were noted to be 5.8 times higher for current smokers and 4.7 times higher for ex-smokers compared to non-smokers [33]. Similarly, a study conducted in the United States reported that current smokers have a fourfold increased risk of PAD compared to non-smokers [37]. In the diabetic population, non-smokers with other indicators of poor lifestyle still had a reduced risk of developing PAD [42].

Lifestyle factors contributing to a poor lifestyle include excessive alcohol intake, poor sleep, and inadequate exercise, which combined increase PAD risk, albeit not independently [42]. Hypertension, defined as a systolic blood pressure of >140 mmHg, is widely mentioned in the literature [32,38,39,43].

Dyslipidemia is another traditional risk factor. A study categorizing it into hypercholesterolemia, hypertriglyceridemia, low high-density lipoprotein (HDL)-cholesterolemia, or mixed hyperlipidemia found a statistically significant prevalence of PAD in populations with dyslipidemia [31,40]. However, it must be noted that when broken down in a study into individual components, only triglyceride levels were independently associated with PAD. These findings are echoed by earlier research, which concluded that serum triglyceride levels were strongly related to PAD, although this has not been consistently replicated in larger epidemiological studies [41]. Low HDL cholesterol may also stand out as a standalone component of dyslipidemia, being shown to be independently associated with PAD in numerous multivariate analyses [31,32,40,43]. Zhu et al. (2024) demonstrated a significant genetic component to the development of PAD among diabetics. They demonstrated that diabetics with a greater genetic risk score, a higher composition of 19 nucleotide polymorphisms linked to PAD, subsequently developed PAD in greater numbers. Obesity as a risk factor for PAD is disputed in the literature, with evidence suggesting that both high and low body mass index (BMI) increase the risk [31,32]. A lower weight-to-hip ratio was found to reduce the risk [32,42]; however, a low BMI of <18 kg/m^2^ conferred a greater risk [38]. These results were consistent across both univariable and multivariable analyses, and the reason remains unclear [41].

The presence of vascular pathologies elsewhere in the body predisposes individuals to develop them in the limbs. Unsurprisingly, individuals with previous coronary artery or cerebrovascular disease have been widely demonstrated to possess a higher risk of PAD [38,39]. The increased risk of chronic kidney disease (CKD) may be less intuitive but is certainly no less important [22,31,38,40]. The pathogenesis of CKD involves inflammatory states contributing to vascular damage. Observed alteration of the extracellular matrix in CKD also contributes to inflammation and increased atherosclerotic damage [38-40]. Another important aspect of the CKD disease process is uremia. High levels of serum uric acid (SUA) have been linked to endothelial dysfunction, oxidative stress, and inflammation, all of which may contribute to the progression of PAD in CKD [44]. However, high SUA, even in the absence of CKD, increases PAD risk [39,43]. Although poorly understood, there exists a strong association between high SUA levels and PAD. It is likely related to high levels of SUA being linked to endothelial dysfunction, oxidative stress, and inflammation, which may all contribute to the disease progression of PAD [44]. Multiple linear regression analysis found this association to be present in the diabetic population, with a higher predicted risk of progression to PAD in individuals with high serum urea [39,43].

Risk factors for peripheral artery disease In diabetic patients unique to Sub-Saharan Africa 

Hayfron-Benjamin et al. found an increased prevalence of macrovascular complications among West African diabetics residing in Africa as opposed to those in the Western world [45]. There is a paucity of research on the reason for these disparities and the risk factors that increase prevalence in Sub-Saharan Africa. Raised C-reactive protein (CRP) was found by Hayfron-Benjamin et al. to be associated with PAD in both diabetics and non-diabetics residing in Sub-Saharan Africa [45]. This was echoed by Mwebaze and Kibirige (2014) stating that hyperglycemia-related inflammation is a hallmark of the pathogenesis of PAD in diabetes [31]. It is, therefore, plausible that a generalized inflammatory state may lead to a greater risk of PAD. Diabetics, in particular, may have more elevated CRP levels due to the proposed hyperglycemia-mediated reduction in immunity and increased rate of chronic infections and infestations [45]. Further, inflammation is a well-known risk factor for the development of atherosclerosis, with elevated CRP levels being strongly linked to the onset of PAD [18]. CRP levels are also abnormally high in individuals with impaired glucose tolerance [22]. Beyond serving as a marker for atherosclerosis, elevated CRP levels may contribute to an increased risk of PAD. CRP exhibits procoagulant properties by promoting the expression of tissue factors and inhibits endothelial nitric oxide (NO) synthase, leading to impaired vascular tone regulation. Additionally, CRP raises levels of plasminogen activator inhibitor-1, which blocks the formation of fibrinolytic plasmin from plasminogen [18,22].

Controversially, Mwebaze and Kibirige (2014) found that females had a higher prevalence of PAD and concluded that the female gender was an independent risk factor [31]. This is disputed by Okello et al. (2014) and Ede et al. (2018) who found no significant difference in the risk between the sexes [32,46]. The study by Mwebaze and Kibirige (2014) had its limitations due to the small sample size and being hospital-based and may, as such, not be generalizable [31]. The literature is similarly divided regarding the importance of age as a risk factor. Agboghoroma et al. (2020) and Umuerri and Obasohan (2013) both concluded that advancing age is significantly associated with the disease [15,29]. However, Okello et al. (2014) and Ede et al. (2018) both found no significant difference [32,46]. This is a clear deviation from the studies conducted in the mostly Western world where advancing age was considered a strong risk factor. There is greater unanimity when it comes to the other traditional risk factors with studies demonstrating dyslipidemia [29,31], and hypertension [15,31,32] as significant risk factors in Sub-Saharan Africa as in the Western world. There is similar agreement in the literature with the duration of diabetes [15,29,46].

Emerging evidence suggests a positive association between HIV infection and the incidence of PAD in African populations. There is a lack of research specifically pertaining to the diabetic population. Kamdem et al. (2018) found that advanced HIV disease was associated with PAD [47]. They proposed that opportunistic infections trigger intense inflammation. Likewise, ecological analyses showed a link between the country-wide prevalence of major endemic infections of HIV, tuberculosis, and malaria and a higher prevalence of HIV. Sickle cell trait was also a proposed risk factor for the disease in the African population as sickle cell trait has been shown to increase vascular dysfunction in diabetics. However, while the study showed an increase in other diabetic complications, it fell short of establishing an association with PAD [47].

Impact of diabetes on peripheral artery disease patients

The clinical course of PAD is significantly influenced by the presence of diabetes, with patient outcomes further modulated by a complex array of comorbidities, including infection, neuropathy, and immune dysfunction. Poor glycemic control has been consistently linked to an elevated prevalence of PAD and an increased risk of adverse events, such as major lower extremity amputation and mortality. Moreover, suboptimal glycemic management is associated with poorer outcomes following revascularization procedures [3]. The clinical course of PAD is significantly more severe in individuals with diabetes compared to those without. Diabetic patients experience a more rapid disease progression, with vascular lesions affecting a wider range of blood vessels. This accelerated disease trajectory is associated with a substantially increased risk of amputation, and there is a significant difference in mortality rates between the two groups [34,48]. The morbidity of PAD includes intermittent claudication, foot ulcers, gangrene, and amputation. The progression of PAD in diabetes is compounded by comorbid conditions such as peripheral neuropathy, which leads to insensitivity of the feet and lower extremities to pain and trauma. With impaired circulation and sensation, ulceration and infection occur, and progression to osteomyelitis and gangrene may necessitate amputation [22]. Furthermore, patients with concomitant diabetes and PAD exhibit a heightened risk of developing ischemic ulcers or gangrene compared to those without diabetes, significantly increasing the likelihood of lower extremity amputation [34].

The Framingham study revealed that diabetes substantially raises the risk of intermittent claudication, with men experiencing a 3.5-fold increase and women an 8.6-fold increase [49]. Diabetic foot ulcers (DFUs) represent a complex interplay of factors, including neuropathy, venous insufficiency, trauma, and infection. PAD significantly contributes to this pathophysiology by exacerbating tissue ischemia, increasing susceptibility to ulceration, and impairing wound healing. These ulcers do not always indicate PAD progression and can occur with adequate peripheral arterial perfusion. Studies show a higher ulcer risk in diabetics with neuropathy and high plantar pressure [50]. Furthermore, individuals with both diabetes and PAD have a significantly increased risk of developing ischemic ulcers or gangrene compared to those without diabetes, thereby elevating the likelihood of lower extremity amputation [51]. Studies show that the prevalence of PAD among individuals diagnosed with DFU ranges from 12% to 55% [52,53]. Chronic limb-threatening ischemia (CLTI) represents the most severe manifestation of PAD, affecting approximately 11% of PAD patients [54]. CLTI carries both an elevated risk of amputation and an elevated risk of cardiovascular morbidity and mortality that varies with CLTI. Studies show that the prevalence of PAD among individuals diagnosed with DFU ranges from 12% to 55% [55]. It has been reported that about 50-70% of all patients with CLTI have DM [56].

DM and PAD are independent risk factors for amputation. However, the coexistence of these conditions synergistically amplifies this risk, with a cohort study demonstrating a fourfold increase in amputation risk compared to the general population. Some older studies have reported that the prevalence of major amputation is significantly elevated in patients with DM, with rates ranging from five to fifteen times higher compared to the non-diabetic population [3].

Multiple studies have also demonstrated poorer prognosis in diabetic PAD patients compared with non-diabetic patients [41,49,57], with mortality occurring at younger ages in diabetics compared to non-diabetics [34]. Patients with both PAD and DM exhibit significantly elevated rates of both fatal and non-fatal cardiovascular and cerebrovascular events compared to those with PAD alone [1]. The disability-adjusted life years (DALYs) are years of healthy life lost representing total disease burden by combining years of life lost and years lived disabled. Of patients with PAD in Sub-Saharan Africa, the DALY score is 20 (UI = 14-30) in males and 17 (UI = 11-26) in females [58]. Hence, for patients with PAD in Sub-Saharan Africa, men lose an average of 20 healthy years of life (range = 14-30 years), and women lose an average of 17 healthy years of life (range = 11-26 years). Amputation as a consequence of DM or PAD imposes a substantial burden on patients, their families, and society. At the individual level, over 55% of patients undergoing amputation due to these conditions experience permanent disability. Moreover, a significant proportion, particularly those undergoing above-knee amputation, are unable to regain ambulatory function [59].

Keller et al. found that diabetic patients with PAD exhibited significantly higher rates of in-hospital mortality (3.5% vs. 2.6%) and major adverse cardiovascular and cerebrovascular events (MACCE) (4.7% vs. 3.3%) compared to non-diabetic counterparts. Diabetic PAD patients also had increased incidences of myocardial infarction, stroke, pulmonary embolism, and pneumonia, whereas deep vein thrombosis was more common in non-diabetic PAD patients. The occurrence of gastrointestinal bleeding and the need for blood transfusions were higher in diabetic PAD patients, but the prevalence of intracerebral bleeding during hospitalization was similar between the groups. DM was identified as an independent predictor of in-hospital mortality and MACCE. Multivariable logistic regression analyses confirmed the significant association of DM with in-hospital death and MACCE, with odds ratios indicating a higher risk in diabetic patients. This association was consistent across both female and male patients [59].

Management of peripheral artery disease in diabetic patients 

Patients with PAD are at a significantly increased risk of both systemic cardiovascular events and limb-related complications. Effective PAD management necessitates a comprehensive approach encompassing lifestyle modifications, such as smoking cessation and regular exercise, in conjunction with optimized medical therapy. Pharmacological interventions for PAD are directed toward reducing the incidence of major cardiovascular events and mitigating limb-related morbidity [60]. Limb-related morbidities are the symptoms and direct effects of impaired blood flow to the leg. These include intermittent claudication, impaired walking due to pain, skin necrosis, and threatened limb viability resulting from chronic critical limb ischemia [61].

The majority of cardiovascular risk factors for individuals with both PAD and diabetes align with those affecting patients with PAD alone. Smoking cessation is the most critical modifiable risk factor for PAD, a fact underscored by its inclusion as a key performance measure in clinical guidelines for PAD management [62]. A meta-analysis by Leng et al. (2000) examining the impact of various lipid-lowering therapies on patients with PAD and dyslipidemia reported a non-significant reduction in mortality and no change in non-fatal cardiovascular events [63]. However, a notable improvement in claudication severity was observed with lipid-lowering treatment [1]. Blood-thinning medications (antiplatelet therapy) for preventing complications in PAD are a double-edged sword.

The Antiplatelet Trialists’ Collaboration conducted a meta-analysis of 145 randomized studies evaluating the efficacy of prolonged antiplatelet therapy, predominantly aspirin. In high-risk patients, a 27% reduction in the composite endpoint of myocardial infarction, stroke, and vascular death was observed compared to controls. However, a subgroup analysis of patients with claudication failed to demonstrate a significant benefit from antiplatelet treatment [64]. The CAPRIE study tested clopidogrel against aspirin in over 19,000 patients with vascular disease [65]. Clopidogrel was slightly more effective than aspirin overall, and significantly more effective for patients with PAD. In summary, current guidelines recommend that patients with diabetes take an antiplatelet agent, such as aspirin or clopidogrel. Those with both diabetes and PAD may find clopidogrel more beneficial [1].

Blood pressure control to a target of ≤140/90 mmHg is recommended for patients with PAD, aligning with general hypertension management. While dedicated trials are limited, compelling evidence supports the benefits of antihypertensive therapy in this population. Angiotensin-converting enzyme inhibitors have shown particular promise, and β-blockers can be safely used when indicated, such as in coronary artery disease [66].

Tight blood sugar control in patients with established PAD may not improve outcomes and might even be harmful. Recent guidelines recommend a personalized approach, considering factors such as age, life expectancy, and existing vascular complications [67]. Stricter targets might be suitable for young, healthy PAD patients, while less stringent control is advised for those with established vascular disease. Several trials have also suggested intensive glucose-lowering treatment might be potentially harmful in patients with established cardiovascular disease. Hyperglycemia is a recognized cardiovascular risk factor, including in patients with PAD. While intensive glycemic control has demonstrated benefits in reducing diabetes-related complications, its direct impact on PAD progression is less established. However, the UK Prospective Diabetes Study (UKPDS) trial demonstrated that there is a significant reduction in diabetes-related endpoints and mortality with intensive glycemic control compared to conventional treatment [1]. Maintaining optimal glycemic control (HbA1c <7.0%) is crucial for preventing microvascular complications in patients with both diabetes and PAD. Individualized treatment plans are essential for managing patients with PAD and diabetes. Patient-specific clinical characteristics should be carefully considered by qualified healthcare professionals when developing optimal treatment strategies [1].

High-intensity statin therapy is a cornerstone in managing symptomatic PAD. Multiple trials have consistently demonstrated the efficacy of lipid-lowering therapy, particularly statins, in improving limb outcomes. These benefits encompass reduced claudication severity, increased walking distance, and decreased risk of chronic and acute limb ischemia, as well as amputation [61]. Regular exercise is important in managing the symptoms of intermittent claudication. Despite the complex underlying mechanisms, exercise training has been shown to alleviate limb symptoms, enhance functional capacity, and reduce systemic cardiovascular risk. Exercise is recommended for all patients with PAD unless contraindicated, such as in the presence of foot ulcers or severe limb pain at rest that requires imminent revascularization [61].

Two pharmacologic agents are approved for the symptomatic management of intermittent claudication in the United States, namely, pentoxifylline and cilostazol. Pentoxifylline, a hemorheological agent, exerts its effects by enhancing red blood cell flexibility and reducing blood viscosity [68]. Cilostazol, a phosphodiesterase inhibitor, is the most efficacious pharmacotherapeutic option currently available for symptomatic intermittent claudication. Clinical trials have demonstrated a substantial improvement in maximal walking distance of 40-50% compared to placebo when cilostazol is administered at a dosage of 100 mg twice daily [18]. In contrast, the efficacy of pentoxifylline in enhancing treadmill walking distance has been modest [69]. Consequently, there is insufficient evidence to support the widespread use of pentoxifylline in PAD [61]. Another medication naftidrofuryl oxalate is an option in certain healthcare systems, such as the United Kingdom, its use is typically reserved for patients who have not responded adequately to supervised exercise and for whom revascularization is contraindicated.

Often medical therapy may not produce optimal outcomes and revascularization is needed. Revascularization has emerged as a critical therapeutic strategy for patients with refractory PAD. Two primary revascularization modalities exist, namely, endovascular interventions and open surgical procedures. Endovascular techniques have experienced substantial growth in recent years, with a more than fivefold increase in utilization reported in the United States between 1980 and 2000 [70]. Endovascular revascularization is generally preferred for patients with localized disease above the knee. While advancements have improved outcomes for long-vessel total occlusions and below-knee interventions, the aortoiliac segment remains the most favorable target for endovascular therapy, with reported one-year patency rates of 80-90% [18]. In contrast, open surgical revascularization, particularly bypass using autogenous vein, often demonstrates superior long-term patency rates in patients with diabetes, especially those with tibial disease [18].

Revascularization is the mainstay of managing patients with critical limb ischemia, aiming to heal ischemic ulcers and prevent limb loss. Surgical revascularization often surpasses endovascular interventions in terms of long-term outcomes. While most ischemic limbs are amenable to revascularization, factors such as target vessel absence, vein graft insufficiency, or irreversible tissue necrosis can preclude this approach. In such cases, amputation may be preferable to prolonged medical treatment [3]. Amputation is indicated in cases of overwhelming, life-threatening infection or when necrosis due to major arterial occlusion has damaged the foot [18]. DM is strongly linked to amputation surgery in PAD patients, particularly minor amputations, but also major amputations, independent of age, sex, and other comorbidities. This correlation was evident in all PAD patients aged 20 years and older [60].

Diabetic foot ulceration is a significant complication in these patients associated with increased all-cause mortality. While endovascular and open surgical approaches have shown comparable limb salvage rates in patients with DFUs and PAD [3], the association between PAD and impaired wound healing is complex. Successful revascularization does not consistently correlate with ulcer healing [71].

Challenges in the management of peripheral artery disease in Sub-Saharan Africa

Sub-Saharan Africa faces several unique challenges in managing PAD among diabetic patients. One of the primary obstacles is the high prevalence of undiagnosed diabetes, with studies indicating that over 54% of people with diabetes in this region remain undiagnosed, the highest proportion globally [6]. This significant barrier is attributed to ineffective screening practices, reduced availability of diagnostic equipment, and a shortage of healthcare workers trained to identify and manage diabetes [12,72,73]. The high cost of treatment, especially insulin, and the limited availability of glucose monitoring equipment hinder access to care, adding pressure on resource-constrained health systems [12]. Additionally, the healthcare infrastructure limitations lead to inadequate screening practices and delayed diagnosis of both diabetes and PAD, resulting in poor health outcomes for affected individuals. For example, in Nigeria, a country with a population of about 200 million, facilities able to provide revascularization procedures are in few major cities and inadequate to meet the population and disease demand [72].

Compounding these challenges, the high prevalence of communicable diseases such as HIV and tuberculosis in Sub-Saharan Africa complicates the management of non-communicable conditions such as diabetes and PAD. The use of ART for HIV is associated with an increased risk of metabolic syndrome, a precursor to type 2 diabetes and cardiovascular disease [11]. This intersection of infectious and non-communicable diseases presents unique public health challenges in the region [10]. Socioeconomic factors and lifestyle changes contribute to the rising prevalence of diabetes and PAD, with economic development leading to changes in nutrition and reduced physical activity [9]. Furthermore, socioeconomic disparities affect access to healthcare and the ability to manage chronic conditions effectively, contributing to high rates of uncontrolled diabetes and subsequent PAD complications. The growing elderly population in Sub-Saharan Africa, associated with higher prevalence rates of PAD among diabetic patients, poses an additional challenge for healthcare systems already strained by limited resources and infrastructure [14,15,74].

Gaps in research

While there is widespread research on DM epidemiology in Sub-Saharan Africa, methodological inconsistencies in study design, diagnostic criteria, and biomarkers hinder comparative analyses, and high-quality data remains limited. Understanding the interplay between diabetes and other prevalent communicable diseases such as HIV and tuberculosis in the region is notably understudied. To effectively address these complex health challenges, concerted efforts are needed to expand knowledge and develop integrated prevention and care programs. Most studies from Sub-Saharan Africa were hospital-based and might not be a reflection of the general population. It is, therefore, important that attention should be given to studies on the general population and in the community. There is no data from controlled, randomized clinical trials available to suggest any benefits from any lifestyle modification dedicated to patients with PAD to reduce the mortality rate and incidence of cardiovascular events; therefore, further research is required.

Summary of key findings

PAD in diabetic patients in Sub-Saharan Africa is prevalent, with rates as high as 61.7% cited in some studies compared to 24% in non-diabetics. DM substantially increases the risk of PAD and its progression, leading to a higher likelihood of ischemic events and impaired functional status among affected individuals. Atherosclerosis, driven by mechanisms such as endothelial dysfunction, inflammation, platelet aggregation, and VSMC dysfunction, underlies the development of PAD in diabetes. The prevalence of diabetes in Sub-Saharan Africa is rising, influenced by economic development, lifestyle changes, and increased life expectancy, with projections indicating a significant increase in diabetic population by 2045. High rates of undiagnosed diabetes, inadequate screening practices, and limited access to diagnosis and treatment contribute to the burden of diabetes and PAD in Sub-Saharan Africa. Risk factors for PAD in diabetic patients include poor glycemic control, smoking, hypertension, dyslipidemia, obesity, advancing age, and the presence of other vascular pathologies or chronic kidney disease. The clinical progression of PAD in diabetic patients is more severe, with higher rates of amputations, ulcers, and infections, and a greater overall mortality risk compared to non-diabetic individuals. Effective management of PAD in diabetic patients involves comprehensive lifestyle modifications, optimal medical therapy, and addressing cardiovascular risk factors, yet these treatments are often underutilized in Sub-Saharan Africa. The intersection of communicable diseases, such as HIV, with diabetes and PAD, poses unique public health challenges, complicating management and increasing the overall disease burden in the region. The healthcare infrastructure in Sub-Saharan Africa is inadequate to meet the needs of the growing diabetic and PAD population, with significant barriers including high treatment costs, limited diagnostic tools, and a shortage of trained healthcare professionals.

Recommendations

Effective management of PAD in diabetic patients in Sub-Saharan Africa requires addressing the high prevalence of undiagnosed diabetes and enhancing access to essential treatments. Implementing comprehensive screening programs and training healthcare workers to diagnose and manage diabetes as well as PAD effectively are critical steps to reduce the burden of PAD. Improving access to affordable insulin, glucose monitoring equipment, and diagnostic tools is essential, especially in rural and underserved areas where the financial cost of treatment is a significant barrier [6,12,73]. Additionally, promoting healthier lifestyles through public health campaigns focused on nutrition, physical activity, and smoking cessation is crucial, as smoking is a significant risk factor for PAD [1,75,76]. Addressing socioeconomic barriers that hinder effective diabetes management, such as healthcare access and affordability, will also help reduce the incidence and severity of PAD in diabetic patients.

Integrating the management of communicable diseases, such as HIV and tuberculosis, with non-communicable diseases, such as diabetes and PAD, can improve overall health outcomes. Given the association between ART for HIV and increased risk of metabolic syndrome and type 2 diabetes, healthcare systems should adopt integrated care models to address both infectious and non-infectious diseases [11]. Investing in healthcare infrastructure to improve the availability and quality of care for PAD patients is vital. Enhancing the capacity of healthcare facilities to provide revascularization procedures and other advanced treatments can significantly impact patient outcomes [77]. By addressing these multidimensional challenges, Sub-Saharan Africa can make substantial progress in managing PAD among diabetic patients, ultimately reducing the burden of this debilitating condition and improving the quality of life for affected individuals.

## Conclusions

PAD is a common complication of diabetes, accelerating disease progression and increasing the risk of cardiovascular and limb morbidity. While neuropathy exacerbates the impact of PAD by reducing foot sensitivity, the diabetic population in Sub-Saharan Africa, ranging from 17% to 61.7%, is particularly vulnerable. Beyond diabetes, traditional PAD risk factors such as obesity, smoking, CKD, hypertension, and dyslipidemia are common. However, the region’s unique epidemiological profile includes additional risk factors such as HIV/AIDS and sickle cell disease. Despite the growing burden of diabetes and PAD, research exploring the interplay between diabetes and other major communicable diseases in Sub-Saharan Africa remains limited. Elevated CRP levels have been linked to an increased incidence of PAD in Sub-Saharan Africa, while the association between sex and PAD is less clear. Patients with PAD are at a heightened risk of both systemic cardiovascular events and limb-related complications. Effective PAD management necessitates a comprehensive approach encompassing lifestyle modifications, such as smoking cessation and regular exercise, in conjunction with optimized medical therapy. It also involves therapy directed toward reducing limb morbidity such as exercise, the use of medications, such as pentoxifylline, cilostazol, and naftidrofuryl oxalate, and revascularization procedures. There is a dearth of interventionists for revascularization procedures in Sub-Saharan Africa.
